# Multifactorial approach and superior treatment efficacy in renal patients with the aid of nurse practitioners.  Design of The MASTERPLAN Study [ISRCTN73187232]

**DOI:** 10.1186/1745-6215-7-8

**Published:** 2006-03-30

**Authors:** Arjan D van Zuilen, Ingeborgh van der Tweel, Peter J Blankestijn, Michiel L Bots, Marjolijn van Buren, Marc AGJ ten Dam, Karin AH Kaasjager, Peter JG van de Ven, Gerald Vervoort, Louis-Jean Vleming, Gerry Ligtenberg, Jack FM Wetzels, the MASTERPLAN study group

**Affiliations:** 1 Department of Nephrology, University Medical Center Utrecht, Utrecht, The Netherlands; 2 Julius Center for Health Sciences and Primary Care, University Medical Center Utrecht, Utrecht, The Netherlands; 3 Dept of Internal Medicine, Haga Hospital, The Hague, The Netherlands; 4 Dept of Internal Medicine, Canisius Wilhelmina Hospital, Nijmegen, The Netherlands; 5 Dept of Internal Medicine, Rijnstate Hospital, Arnhem, The Netherlands; 6 Dept of Internal Medicine, Medical Center Rijnmond Zuid, Rotterdam, The Netherlands; 7 Department of Nephrology, Radboud University Nijmegen Medical Center, Nijmegen, The Netherlands

## Abstract

**Background:**

Patients with chronic kidney disease (CKD) are at a greatly increased risk of developing cardiovascular disease. Recently developed guidelines address multiple risk factors and life-style interventions. However, in current practice few patients reach their targets.

A multifactorial approach with the aid of nurse practitioners was effective in achieving treatment goals and reducing vascular events in patients with diabetes mellitus and in patients with heart failure. We propose that this also holds for the CKD population.

**Design:**

MASTERPLAN is a multicenter randomized controlled clinical trial designed to evaluate whether a multifactorial approach with the aid of nurse-practicioners reduces cardiovascular risk in patients with CKD. Approximately 800 patients with a creatinine clearance (estimated by Cockcroft-Gault) between 20 to 70 ml/min, will be included. To all patients the same set of guidelines will be applied and specific cardioprotective medication will be prescribed. In the intervention group the nurse practitioner will provide lifestyle advice and actively address treatment goals. Follow-up will be five years. Primary endpoint is the composite of myocardial infarction, stroke and cardiovascular mortality. Secondary endpoints are cardiovascular morbidity, overall mortality, decline of renal function, change in markers of vascular damage and change in quality of life. Enrollment has started in April 2004 and the study is on track with 700 patients included on October 15th, 2005. This article describes the design of the MASTERPLAN study.

## Background

Patients with chronic kidney disease are at a greatly increased risk of developing cardiovascular disease (CVD)[1,2]. This is most prominent in patients on renal replacement therapy but also firmly established in patients with mild renal dysfunction[3]. This increased cardiovascular risk in patients with chronic kidney disease (CKD) is the resultant of a multitude of risk factors. Among these risk factors are: all known traditional risk factors, a number of them evidently more prevalent than in the general population, risk factors that are associated with or worsened by renal insufficiency (anaemia, disturbances in calcium-phosphorusbalance, oxidative stress, inflammation) and renal insufficiency itself [4-7].

Several guidelines have been formulated, both nationally and internationally, to assist physicians in adequately reducing cardiovascular risk [8-11]. However it is well known that patients do not reach treatment-goals formulated in these guidelines[12]. This has also been established in patients with renal disease[13,14]. In patients with diabetes mellitus and heart failure a multifactorial intervention implemented by nurse-practitioners significantly improved metabolic control and reduced cardiovascular events [15-18].

Given the high cardiovascular risk and the multitude of modifiable risk factors a multifactorial approach could very well also be of benefit for patients with CKD. This has been suggested several times but has never been proven. [4,19,20] Most risk factors that promote CVD also promote decline of renal function. Effectively addressing these risk factors might therefore also delay renal function decline[21,22].

## Design

The MASTERPLAN study is designed as a multicenter randomized controlled clinical trial with a duration of 5 years of follow-up.

### Aims & primary outcome

The MASTERPLAN study aims to show that in patients with mild to moderate CKD strict implementation of current guidelines by a nurse practitioner, with emphasis on the use of cardioprotective medication and lifestyle changes, results in a reduction of a composite endpoint of myocardial infarction, stroke and cardiovascular mortality.

Cardiovascular morbidity, overall mortality, quality of life, percentage of patients achieving treatment goals, changes in renal function and changes in markers of vascular damage will be considered as secondary outcomes.

### Measurements

Changes in renal function will be documented by annually measuring creatinine clearance from a 24-hour urine sample and measurement of the serum creatinine. Quality of life will be assessed through questionnaires that will be filled out by all participants annually. Markers of vascular damage will be recorded annually in a proportion of the patients. These markers address different aspects like endothelial function, arterial compliance and atherosclerosis in various vascular beds. Pulse wave analysis (PWA) and pulse wave velocity (PWV) will be measured in approximately 300 patients. Aortic pulse wave forms and aortic pulse wave velocity are indicators of arterial stiffness. PWV has been shown to be an independent predictor for cardiovascular risk[23,24]. PWA and PWV measurements have been validated and can be measured in a reproducible manner[25]. Carotid intima media thickness (CIMT) evaluation through B-mode sonography will also be measured in approximately 150 patients. Increased CIMT is associated with an elevated risk of cardiovascular disease[24]. Change in CIMT has been shown to result from risk factor modifications in a variety of populations[26]. CIMT measurement has been validated and shown to be reproducible[27].

Blood pressure will be recorded twice per year using a non-invasive automated oscillometric device. Patients will stay in a supine position for 30 minutes, blood pressure will be recorded every three minutes. The last five measurements will be used for analysis. Conventional office readings using the auscullatory method will also be documented.

### Recruitment and screening

All subjects will be recruited from the outpatient nephrology or internal medicine clinic of nine Dutch hospitals. Patients are identified by checking their medical records for compatibility with the inclusion criteria prior to a regular outpatient visit. Renal function will be estimated using the Cockcroft-Gault formula (with a correction for body surface area as of April 15^th ^2005)(appendix A). The Cockcroft-Gault equation has been extensively validated and is generally appreciated as a useful tool to estimate creatinine clearance[28,29]. The physician will give every eligible patient a brief verbal explanation and extensive written information about the study. A week later follow-up by the nurse practitioner will be performed. Upon verbal consent to participate in the study, the patient will be invited to the office. In this visit the in- and exclusion criteria will be checked thoroughly, written informed consent will be acquired and subsequently baseline measurements will be performed. The medical ethics committees of the participating hospitals have approved the conduct of the study.

Recruitment began in April 2004 and is estimated to continue until December 2005. About 60% of patients deemed eligible by their physician and asked to participate in the study, actually participate and are included. The main reasons for non-participation appear to be: reluctance of the patient to changes in drug therapy and inability of the patient to attend the required visits. On December15, 2005 793 patients had been included.

### In- and exclusion criteria

Patients are eligible for inclusion when they fulfill the following criteria:

• The subject is at least 18 years old

• The subject is diagnosed with CKD with a creatinine clearance estimated by the Cockcroft-Gault equation between 20 and 70 ml/min.

• The subject is able and willing to provide written informed consent

The following conditions are considered exclusion criteria:

• A renal transplant less than a year before inclusion

• Acute renal failure or rapidly progressive glomerulonephritis established by the treating physician

• Any malignancy less than five years before inclusion other than basocellular or squamous cell carcinoma of the skin.

• Participation in other clinical trials requiring the use of study medication

From April 15^th ^2005 until the end of the inclusion the Cockcroft-Gault equation was modified to take into account body surface area according to recent insights into the applicability of formulas to estimate renal function [30-33]. This modification was approved by the medical ethics committee.

### Baseline evaluation and randomization

Table 1 summarizes the data collected at baseline evaluation. After the baseline evaluation, the patient will be randomized to either nurse practitioner care or physician care. Randomization to treatment is stratified by center and renal transplant status using a web-based randomization module and performed in predefined blocks of certain numbers of patients.

**Table 1:** Data collection

Visit	Baseline 1	Visit 2	Visit 3	Visit 4	Visit 5
Month	0	3	6	9	12

Informed Consent	x				
Demographic data	x				
Medical history	x				
In- and exclusion criteria	x				
Randomization	x				
Endpoints		x	x	x	x
Weight	x	x	x	x	x
Waist-hip ratio	x				x
Ankle-Brachial Index	x				x
RR and HR in sitting position	x	x	x	x	x
RR 30 minutes Dynamap	x		x		x
Smoking habit	x	x	x	x	x
Medication use	x	x	x	x	x
Three monthly Laboratory evaluation^a^	x	x	x	x	x
Annual Laboratory evaluation ^b^	x				x
ECG	x				x
PWV (Proportion of patients)	x				x
Carotid-IMT (Proportion of patients)	x				x
Questionnaires ^c^	x				x

a: Three monthly Laboratory evaluation: Hematology: Hb, Ht, trombocyte count; Chemistry: sodium, potassium, calcium, phosphate, bicarbonate, urea, creatinine, albumin; Urinalysis: spot urine creatinine, albumin, total protein

b: Annual Laboratory evaluation: Hba1c, uric acid, PTH, total-cholesterol, HDL-cholesterol, LDL-cholesterol, triglycerides; Urinalysis: 24 hr urine sodium, creatinine, albumin, total protein

c: Questionnaires: Quality of Life: SF-36, EQ-5D; Physical activity: SQUASH; Dietary composition; Erectile dysfunction

### Treatment groups

To all patients the same set of guidelines and treatment goals, represented in Table 2 and Table 3, apply. Both patients and physicians are provided with information about the beneficial effects of multifactorial risk factor management regardless of treatment allocation. In the intervention group nurse practitioners, supervised by a qualified nephrologist, will actively pursue lifestyle intervention (physical exercise, nutritional counseling, weight reduction and smoking cessation), the use of specified cardioprotective medication and the implementation of current guidelines. The nurse practitioner will check regularly whether treatment goals have been met and when deemed appropriate adjust treatment to achieve target values. Modification of therapy will be executed according to flowcharts that have been derived from current guidelines.

**Table 2:** Goals and relevant guidelines for cardiovascular risk factors in MASTERPLAN

Risk factors	Goal	Guideline
Blood pressure	</= 130/85 mm Hg^a^	NFN^b^8, KDOQI^c^[10]
Proteinuria Protein excretion in urine	< 0.5 g/day	KDOQI^c^[10]
Lipids		KDOQI^c^[11,44]
Fasting LDL	< 2.6 mmol/l	
Anemia		NFN^b^[45],
Hemoglobin concentration	> 6.8 mmol/l	KDOQI^c^[46]
Glucose		NHG^d ^[47]
Fasting glucose	< 7.0 mmol/l	
Non Fasting glucose	< 9.0 mmol/l	
Calcium and Phosphate metabolism		NFN^b^[8],
Phosphate	</= 1.8 mmol/l	KDOQI^c^[48]
PTH	< 18 pMol/l**	
Healthy Nutrition		GR^f^[49]
Protein	0.8–1.0 g/kg ideal bodyweight/day	
Sodium	2000 mg/day	
Fat	Reduce fat, unsaturated fats preferred	
Energy	30–35 kcal/kg ideal bodyweight/day	
Overweight Body mass Index	<25 kg/m^2^	
Physical exercise	5×/week 30 minutes moderate exercise	NNGB^g^[50]
Smoking	To Quit	NFN^b^[8]

a In case of proteinuria > 1 g/day: 125/75 mm Hg

b NFN: Nederlandse Federatie voor Nefrologie (Dutch Federation for Nephrology)

c KDOQI: Kidney Disease Outcomes Quality Initiative

d NHG: Nederlands huisartsen genootschap (Dutch College of General Practitioners)

e smaller than three times the upper limit of the normal range f GR: Gezondheidsraad (Health Council of the Netherlands)

g NNGB: Nederlandse Norm voor Gezond Bewegen (Dutch Standard of Healthful Physical Activity)

**Table 3:** Standard medication to reduce cardiovascular risk in MASTERPLAN

Medication	Recommended dose	Point of impact
HMG-CoA Reductase Inhibitor (statin)	e.g. atorvastatin 10 mg daily (or comparable dose of other statin)	Lipid-metabolism
Acetylsalicylic acid	80 mg daily	Thrombocyte aggregation
Folic Acid	5 mg daily	Homocysteine metabolism
ACE inhibitor or Angiotensin Receptor Blocker (ARB)	e.g. enalapril 5 mg twice daily (or comparable dose of other ACE inhibitor) or irbesartan 75–150 mg (or comparable dose of other ARB) daily	Blood pressure, renal function and cardiac pre- and afterload

a In case of proteinuria > 1 g/day: 125/75 mm Hg b NFN: Nederlandse Federatie voor Nefrologie (Dutch Federation for Nephrology) c KDOQI: Kidney Disease Outcomes Quality Initiative d NHG: Nederlands huisartsen genootschap (Dutch College of General Practitioners) e smaller than three times the upper limit of the normal range f GR: Gezondheidsraad (Health Council of the Netherlands) g NNGB: Nederlandse Norm voor Gezond Bewegen (Dutch Standard of Healthful Physical Activity)

Physician care comprises 'usual care' conform the guidelines mentioned in Table 2. In contrast to the intervention group and in agreement with real life practice no extra incentives to adhere to the guidelines will be supplied.

### Clinical data

The intervention group will receive follow up by the nurse practitioner as often as is indicated by the guidelines and the sort of lifestyle intervention the patient receives. Additional data will be collected for trial purposes quarterly as described in table 1. The physician care group will receive an automated non-invasive blood pressure measurement two times a year. Annually each patient will be invited in the office to undergo a series of measurements and to fill out questionnaires. At the other time-points (represented in Table 1) present clinical data derived from the medical records will be recorded in the case report form.

The patients will fill out several questionnaires. Firstly quality of life will be assessed using the SF-36 (Dutch version) and EQ-5 D (Dutch version) questionnaires[34,35]. Secondly the Short Questionnaire to asses health enhancing physical activity will be given to subjects[36]. Thirdly a Food questionnaire developed by the Wageningen University will deliver information about the composition of the diet of the subjects[37].

### Data management

The entire study is performed according to the ICH-GCP guidelines, including on-site monitoring. The data will be documented in a case report form, via a validated web-based structure. Investigators will fill out the required data of a visit in an Internet application based on PDF files. Upon completing the form they will start the submission process. Before submission the data are verified and passed through an editing process to check for logical inconsistencies within the data. Any discrepancies need to be corrected before actual submission can take place. The data will be sent to a web server located in the data management center. The data are immediately transferred into a SQL server database.

The questionnaires are all in Teleform format and mailed to the data management center. Filled out questionnaires are scanned and data are immediately transferred to the same database used to store the clinical data. A specified validation procedure has been developed to check inconsistencies and to generate and process queries.

### Endpoint collection and evaluation

The primary endpoint has been defined as follows, based on experience in other studies run at the UMCU [38,39].

• Myocardial infarction is defined as evident new ischemic changes on an ECG or an established rise and fall pattern of cardiac enzymes.

• Stroke is defined as characteristic clinical symptoms of stroke accompanied by signs of recent ischemia using an appropriate imaging technique (CT-scan or MRI).

• Cardiovascular mortality is defined as death due to myocardial infarction, stroke, ruptured abdominal aneurysm, and terminal heart failure. Also sudden death will be regarded as part of cardiovascular mortality.

All suspected events, which might contribute to the primary endpoint, will be evaluated by an independent endpoint-committee. The committee will evaluate these events using definitions for the different events which are also used in other Dutch trials [38,39].

Secondary endpoints are collected via clinical data, questionnaires or specified procedures. The SphygmoCor system (pulse wave velocity system and blood pressure analysis system, PWV Inc., Sydney Australia) is used to assess PWV and to analyze the arterial pulse contours. Pulse contours will be obtained by applanation tonometry at the carotid, radial and femoral arteries. Independent investigators blinded for treatment allocation of the subject will perform evaluation of recorded data. CIMT assessment will be performed using B-mode ultrasonography of both carotid arteries with a 7,5-MHz linear array transducer to assess the presence of plaques in the common carotid artery, bifurcation and internal carotid artery. All measurements will be recorded on video and evaluated off-line by independent evaluators blinded for treatment allocation of the patient [40].

### Nurse practitioners

The nurse practitioners are all qualified nurses with several years of experience and affinity for nephrology. They received extensive education in cardiovascular risk reduction with emphasis on current guidelines, they were familiarized with the contents of the flowcharts and all were uniformly trained in interview techniques to support them in implementing lifestyle-modification and maximizing compliance. Completion of the official Dutch Nurse Practitioner training program was no prerequisite to function as a nurse practitioner in this study.

### Statistical and data analysis

#### Power analysis

To detect a 50% between-group difference in the trial primary endpoint with a pre-estimated 5-year event incidence of 13.5% for the control group [16,41], a power of 80% and a two-sided type I error α of 5%, at least 640 patients will have to be included. Taking into account a possible loss to follow-up of 15%, at least 740 patients will be randomized.

#### Group sequential analysis

Group sequential analysis will be used to evaluate the primary endpoints and to monitor the safety data. Sequential analysis is a statistical approach where one conducts significance tests over time as the data are collected. Sequential analysis and its application in clinical trials has been described extensively by Whitehead [42]. The general approach is as follows. A null hypothesis H_0 _and an alternative hypothesis H_1 _are formulated for a suitable measure θ of treatment difference. For this study with a survival type outcome variable, θ is equal to the logarithm of the hazard ratio (HR). H_0 _is formulated as "no difference in the occurrence of the primary endpoints between the two trial arms" or θ = 0 (i.e. HR = 1). The alternative hypothesis H_1 _is formulated as |θ | ≥ log(0.48) = 0.73 group. Two test statistics, Z and V, can be derived depending on the type of response variable. Z is a measure of the treatment difference; for survival data Z is the observed number of events in the control group minus the expected number of events given treatment equivalence (i.e. the number of events that would have occurred if the same proportion of events was found in the intervention group and in the control group).V reflects the amount of information about θ contained in Z; for survival data V is approximately equal to a quarter of the number of events observed. The sequential analysis requires critical boundaries to be specified in advance. These boundaries depend onθ, the type I error α and the power 1-β. For each new group of patients, values of Z and V are calculated and presented graphically by plotting Z against V (see Figure1 for an illustration of a double-sided sequential test). Based on the path of cumulative (Z, V)-points, one of the following three decisions is made:

1) the upper or the lower (continuous) boundary is crossed: stop the data collection and reject the null hypothesis;

2) one of the inner wedge-shaped (dashed) boundaries is crossed: stop the data collection and accept the null hypothesis;

3) continue the data collection: the cumulative data are inadequate to draw a conclusion yet.

With a sequential approach a trial can be stopped earlier, on average, than with a fixed sample size approach. Using a sequential design between 460 and 716 patients would have to be included. (The number of patients to be included can not be fixed beforehand because patient data are analyzed cumulatively and a decision is made to stop or to continue the trial according to the interim results.) Due to the large difference between the duration of the inclusion period and that of the follow-up, it will not be possible to implement this potential benefit in the design of the trial. Although the length of follow-up in this trial in relation to the period scheduled for inclusion does not allow for a reduction in sample size other benefits of group sequential analysis remain present. Earlier clarity on the primary endpoint could allow for earlier stopping of the trial and thus result in saving time and funds for other experiments. Also safety related issues, which are to be monitored one-sided, will possibly be identified earlier and in this way patient safety is guaranteed throughout the trial.

### Statistical methods

Group sequential (or interim) analyses will be performed using the double triangular test as described by Whitehead [42] and implemented in the computer program PEST version 4 [43]. The analyses will be performed by an independent data safety monitoring board (DSMB) consisting of a nephrologist, an internist and a statistician. They meet every 6 months to monitor various aspects of the study, including recruitment, adverse events and interim analyses of the primary endpoints.

The results of the study will be analyzed following the 'intention to treat' principle. This means that subjects will be analyzed according to the group they have been allocated to by the randomization. Results will be presented as Kaplan-Meier curves for the two treatments and the difference between the treatments will be analyzed using a log-rank test. For the primary outcome variables the log-rank test will be adjusted for the effect of the cumulative data analyses. Results will be presented for all cause mortality and CV events separately.

The primary analysis of CIMT progression wiw ll be performed using a linear random coefficient (Laird-Ware) model using real visit days, treatment and clinical center as independent variables. For each participant, the intercept and slope of CIMT change over time is assumed to be a normally distributed random variable with different means for the two treatment groups. The mean slope for the nurse practitioner group will be compared to that for nephrologist-care group using linear contrasts and a 5% significance level. Additional exploratory analyses will evaluate the impact of including baseline IMT, lumen diameter, ultrasound reader, and center as additional co-variates.

The data analytic approach to arrive at the PWV outcome variable is similar to that of the CIMT outcome. Adjustments that will be taken into account in the estimates are changes in MAP and changes in heart rate, since both are closely related to PWV.

## Discussion

### Limitations of the study

There are several limitations to the study mostly due to unavoidable decisions.

1. Although many patients receiving standard care are not treated according to current guidelines it is ethically not possible to withhold information and advice on these guidelines from the control group. Therefore all patients receive information about risk factor management and all physicians are informed about the treatment goals. This may result in improved treatment in the control group thereby attenuating the potential difference in cardiovascular events between the two treatment arms.

2. Due to the sort of intervention blinding is not possible for patients or physicians. This is unavoidable since some physicians will be treating or supervising both physician care and nurse practitioner care patients. Again, attenuation of group differences may occur.

3. The multifactorial intervention will make it impossible to single out one aspect of the intervention as being the most beneficial. Since trials establishing the exact amount of risk reduction per risk factor intervention are missing in patients with mild to moderate renal dysfunction, such information would have been most useful. As pointed out earlier however the choice for a multifactorial design has received ample consideration. As a consequence of this choice for a multifactorial approach sample size is too small to allow for a definite statement about a single risk factor. A study large enough to establish the benefits of one aspect of the intervention would require thousands of patients and the logistics and funding necessary to realize this are not present for investigator driven research.

## Conclusion

Cardiovascular risk in patients with CKD is very high, multifactorial in origin and present early in the course of CKD. Effectively addressing risk factors will reduce cardiovascular risk significantly. A multifactorial approach with the aid of nurse practitioners has been shown to be effective in other high-risk populations. The MASTERPLAN trial is designed to establish the effects of such a multifactorial approach in patients with mild to moderate renal insufficiency. The MASTERPLAN trial is a unique multicenter randomized clinical trial because it investigates the effects of a multifactorial approach to reduce cardiovascular events in a population until now rarely targeted despite a huge cardiovascular risk. The employment of the nurse practitioner provides a valuable means of implementing the multifactorial intervention.

## Appendix A

The Cockcroft-Gault equation



The Cockcroft-Gault equation modified to correct for body surface area (effective from april 15^th ^2005).



Body Surface Area will be estimated using the formule by Dubois and Dubois.

## Appendix B

### Masterplan study group

Peter J. Blankestijn

Michiel L. Bots

Marjolijn van Buren

Marc A.G.J. ten Dam

Karin A.H. Kaasjager

Gerry Ligtenberg

Yvo W. Sijpkens

Henk E. Sluiter

Peter J. van de Ven

Gerald Vervoort

Louis-Jean Vleming

Jack F.M. Wetzels

Arjan D. van Zuilen

### Participating centers

• Canisius Wilhelmina Hospital, Nijmegen, The Netherlands

• Deventer Hospital, Deventer, The Netherlands  

• Haga Hospital, Location Leyenburg, The Hague, The Netherlands

• Haga Hospital, Location Rode Kruis, The Hague, The Netherlands

• Leiden University Medical Center, Leiden, The Netherlands

• Medical Center Rijnmond Zuid, Rotterdam, The Netherlands

• Rijnstate Hospital, Arnhem, The Netherlands

• Radboud University Nijmegen Medical Center, Nijmegen, The Netherlands

• University Medical Center Utrecht, Utrecht, The Netherlands

### Event committee

• Dr. J.J. Beutler, nephrologist, Jeroen Bosch Hospital, 's Hertogenbosch, The Netherlands, (chair)

• Dr.J.D. Banga, internist, University Medical Center Utrecht, Utrecht, The Netherlands,

• Dr. A.P. Van Dijk, cardiologist, Radboud University Nijmegen Medical Center, Nijmegen, The Netherlands,

• Dr. J.W.M. Keunen, neurologist, Haga Hospital, Location Leyenburg, The Hague, The Netherlands,

### Data safety and monitoring board

• dr. I. van der Tweel, department of biostatics, Julius Center for Health Sciences and Primary Care, University Medical Center Utrecht, Utrecht, The Netherlands (chair)

• prof. dr. J.W. Lenders, department of internal medicine, Radboud University Nijmegen Medical Center, Nijmegen, The Netherlands

• prof. Dr. T.J. Rabelink, department of nephrology, Leiden University Medical Center, Leiden, The Netherlands

### Data management center

Julius Center for Health Sciences and Primary care, UMC Utrecht, Utrecht, The Netherlands

## Authors' contributions

AvZ drafted the manuscript, IvdT contributed extensively to the statistical analysis section. GL was the initiator of the study. PB, MB, MtD, MvB, PvdV, GV, LV and JW contributed to the design of the study. PB, MB, GV, GL, and JW helped to draft the manuscript. All authors read and approved the final manuscript. The authors declare that they have no competing interests.
